# Dichlorido{*N*-[1-(pyrazin-2-yl)ethyl­idene-κ*N* ^1^]ethane-1,2-diamine-κ^2^ *N*,*N*′}zinc

**DOI:** 10.1107/S1600536811042115

**Published:** 2011-10-22

**Authors:** Jia-Cheng Liu, Meng Li, Abedelwahed Saeed Mohammed Omer, Yun Wei, Guo-Zhe Guo

**Affiliations:** a College of Chemistry and Chemical Engineering, Key Laboratory of Eco-Environment-Related Polymer Materials of the Ministry of Education, Key Laboratory of Polymer Materials of Gansu Province, Key Laboratory of Bioelectrochemistry & Environmental Analysis of Gansu, Northwest Normal University, Lanzhou 730070, People’s Republic of China

## Abstract

The Zn^II^ atom in the title complex, [ZnCl_2_(C_8_H_12_N_4_)], is coordinated by two Cl atoms and three N atoms of the *N*-[1-(pyrazin-2-yl)ethyl­idene]ethane-1,2-diamine ligand, and displays a distorted square-pyramidal geometry with the apical position occupied by a Cl atom. In the crystal, inter­molecular N—H⋯Cl and C—H⋯Cl hydrogen bonds link the mol­ecules into a three-dimensional framework.

## Related literature

For the use of dinucleating *N*-heterocyclic ligands in crystal engineering, see: Pascu *et al.* (2004 ▶). For metal complexes of Schiff base ligands in coordination chemictry, see: Coles *et al.* (1998 ▶); Gourbatsis *et al.* (1999 ▶).
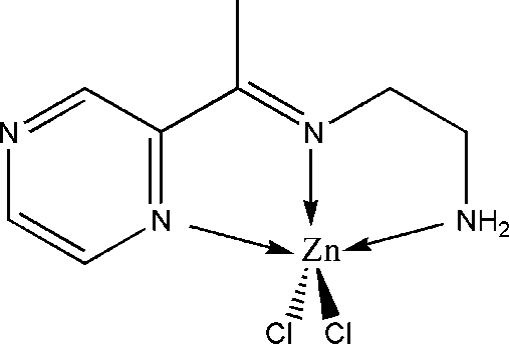


## Experimental

### 

#### Crystal data

[ZnCl_2_(C_8_H_12_N_4_)]
*M* *_r_* = 300.49
Triclinic, 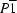

*a* = 7.106 (5) Å
*b* = 8.976 (6) Å
*c* = 10.225 (6) Å
α = 69.566 (5)°
β = 73.434 (5)°
γ = 83.056 (5)°
*V* = 585.6 (6) Å^3^
*Z* = 2
Mo *K*α radiation
μ = 2.53 mm^−1^
*T* = 296 K
0.23 × 0.21 × 0.19 mm

#### Data collection

Bruker APEXII CCD diffractometer
Absorption correction: multi-scan (*SADABS*; Bruker, 2008 ▶) *T*_min_ = 0.594, *T*_max_ = 0.646
4159 measured reflections
2129 independent reflections
1790 reflections with *I* > 2σ(*I*)
*R*_int_ = 0.027

#### Refinement

*R*[*F* ^2^ > 2σ(*F* ^2^)] = 0.031
*wR*(*F* ^2^) = 0.071
*S* = 1.08
2129 reflections
137 parameters
H-atom parameters constrained
Δρ_max_ = 0.31 e Å^−3^
Δρ_min_ = −0.38 e Å^−3^

### 

Data collection: *APEX2* (Bruker, 2008 ▶); cell refinement: *SAINT* (Bruker, 2008 ▶); data reduction: *SAINT*; program(s) used to solve structure: *SHELXS97* (Sheldrick, 2008 ▶); program(s) used to refine structure: *SHELXL97* (Sheldrick, 2008 ▶); molecular graphics: *SHELXTL* (Sheldrick, 2008 ▶); software used to prepare material for publication: *SHELXTL*.

## Supplementary Material

Crystal structure: contains datablock(s) I, global. DOI: 10.1107/S1600536811042115/bh2386sup1.cif

Structure factors: contains datablock(s) I. DOI: 10.1107/S1600536811042115/bh2386Isup2.hkl

Additional supplementary materials:  crystallographic information; 3D view; checkCIF report

## Figures and Tables

**Table 1:** Hydrogen-bond geometry (Å, °)

*D*—H⋯*A*	*D*—H	H⋯*A*	*D*⋯*A*	*D*—H⋯*A*
N4—H4*B*⋯Cl1^i^	0.90	2.77	3.531 (4)	143
N4—H4*A*⋯Cl2^ii^	0.90	2.81	3.534 (3)	139
C2—H2⋯Cl2^iii^	0.93	2.81	3.672 (4)	155
C3—H3⋯Cl2^iv^	0.93	2.80	3.704 (4)	164
C6—H6*B*⋯Cl1^i^	0.97	2.84	3.550 (4)	131

Symmetry codes: (i) 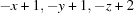; (ii) 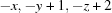; (iii) 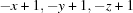; (iv) 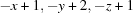.

**Fig. 1.**
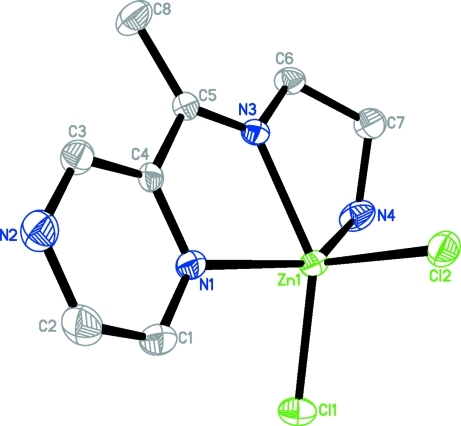
The structure of the title compound. Displacement ellipsoids are drawn at the 30% probability level. H atoms have been omitted for clarity.

**Fig. 2.**
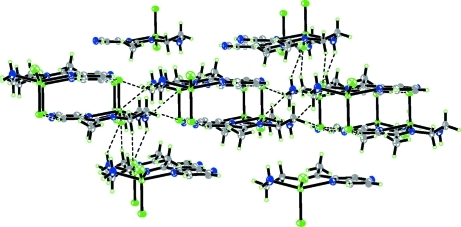
The crystal packing of the title compound, viewed along the b axis. Hydrogen bonds are shown as dashed lines.

## supplementary crystallographic information

### Comment

Pyrazines themselves are well known dinucleating ligands and, as many dinucleating *N*-heterocyclic ligands, have attracted much attention from crystal engineers (Pascu *et al.*, 2004). On the other hand, Schiff base ligands have played an integral role in the development of coordination chemistry since the late 19 th century. The finding that metal complexes of these ligands are ubiquitous reflects their facile synthesis, wide applications and the accessibility to diverse structural modifications (Coles *et al.*, 1998; Gourbatsis *et al.*, 1999). Herein, we report on the synthesis of an asymmetric Schiff base using 2-acetylpyrazine as precursor and we report its Zn^II^ complex.

The molecular structure of the complex [ZnCl_2_(C_8_H_12_N_4_)] is shown in Fig. 1. The complex is a mononuclear, five-coordinate species. The central zinc ion is coordinated by two chloride and three N atoms. The Schiff base acts as a tridentate chelating ligand, giving two five-membered rings. The coordination geometry about zinc(II) is distorted square-pyramidal. Atoms N1, N3, N4 and Cl1 form the basal plane and atom Cl2 is in apical position. The effect of the chelate rings is clearly observed in the N1—Zn1—N3 and N3—Zn1—N4 bond angles, which deviate by 16.7 and 12.0°, respectively, from the ideal value (90°). As a result, the N1—Zn1—N4 axis is not linear [145.43 (10)°], significantly deviated from the ideal value of 180°. The Zn—N distances in the basal plane are 2.122 (3), 2.245 (2), and 2.117 (3) Å.

In the crystal packing (Fig. 2), N—H···Cl and C—H···Cl hydrogen bonds link the molecules into sheets, which interact weakly to form a 3D framework.

### Experimental

To 0.2 mmol (0.0328 g) of *N*-[1-(pyrazin-2-yl)ethylidene]ethane-1,2-diamine in 15 ml of methanol was added 0.2 mmol (0.0272 g) of ZnCl_2_ in 10 ml of methanol, and the mixture was stirred at 333 K for 0.5 h. Upon free evaporation, single crystals suitable for XRD analysis were collected by filtration within two weeks (yield: 69%).

### Refinement

All H atoms were included in calculated positions, with C—H distances ranging from 0.93 to 0.97 Å, and N—H distances of 0.90 Å. They were refined in the riding-model approximation, with *U*_iso_(H) = 1.2*U*_eq_(carrier C,N) or *U*_iso_(H) = 1.5*U*_eq_(C methyl).

### Crystal data

**Table:**

[ZnCl_2_(C_8_H_12_N_4_)]	*Z* = 2
*M**_r_* = 300.49	*F*(000) = 304
Triclinic, *P*1	*D*_x_ = 1.704 Mg m^−^^3^
Hall symbol: -P 1	Mo *K*α radiation, λ = 0.71073 Å
*a* = 7.106 (5) Å	Cell parameters from 1862 reflections
*b* = 8.976 (6) Å	θ = 2.2–26.4°
*c* = 10.225 (6) Å	µ = 2.53 mm^−^^1^
α = 69.566 (5)°	*T* = 296 K
β = 73.434 (5)°	Block, colourless
γ = 83.056 (5)°	0.23 × 0.21 × 0.19 mm
*V* = 585.6 (6) Å^3^	

### Data collection

**Table:**

Bruker APEXII CCD diffractometer	2129 independent reflections
Radiation source: fine-focus sealed tube	1790 reflections with *I* > 2σ(*I*)
graphite	*R*_int_ = 0.027
φ and ω scans	θ_max_ = 25.5°, θ_min_ = 2.2°
Absorption correction: multi-scan (*SADABS*; Bruker, 2008)	*h* = −8→8
*T*_min_ = 0.594, *T*_max_ = 0.646	*k* = −9→10
4159 measured reflections	*l* = −12→12

### Refinement

**Table:**

Refinement on *F*^2^	Primary atom site location: structure-invariant direct methods
Least-squares matrix: full	Secondary atom site location: difference Fourier map
*R*[*F*^2^ > 2σ(*F*^2^)] = 0.031	Hydrogen site location: inferred from neighbouring sites
*wR*(*F*^2^) = 0.071	H-atom parameters constrained
*S* = 1.08	*w* = 1/[σ^2^(*F*_o_^2^) + (0.032*P*)^2^] where *P* = (*F*_o_^2^ + 2*F*_c_^2^)/3
2129 reflections	(Δ/σ)_max_ = 0.001
137 parameters	Δρ_max_ = 0.31 e Å^−^^3^
0 restraints	Δρ_min_ = −0.38 e Å^−^^3^
0 constraints	

### Fractional atomic coordinates and isotropic or equivalent isotropic displacement parameters (Å^2^)

**Table:**

	*x*	*y*	*z*	*U*_iso_*/*U*_eq_	
Zn1	0.31700 (5)	0.62496 (4)	0.82328 (3)	0.03108 (13)	
C1	0.6706 (5)	0.5945 (4)	0.5517 (3)	0.0429 (8)	
H1	0.6344	0.4897	0.5783	0.051*	
C2	0.8103 (5)	0.6600 (4)	0.4227 (3)	0.0478 (9)	
H2	0.8639	0.5976	0.3645	0.057*	
C3	0.7916 (4)	0.8893 (4)	0.4693 (3)	0.0348 (7)	
H3	0.8318	0.9926	0.4442	0.042*	
C4	0.6524 (4)	0.8264 (3)	0.5984 (3)	0.0271 (6)	
C5	0.5617 (4)	0.9145 (3)	0.7031 (3)	0.0281 (6)	
C6	0.3104 (4)	0.9118 (3)	0.9186 (3)	0.0343 (7)	
H6A	0.2748	1.0230	0.8787	0.041*	
H6B	0.3952	0.9032	0.9804	0.041*	
C7	0.1281 (5)	0.8162 (4)	1.0050 (3)	0.0425 (8)	
H7A	0.0788	0.8336	1.0974	0.051*	
H7B	0.0269	0.8504	0.9533	0.051*	
C8	0.6519 (5)	1.0628 (4)	0.6874 (4)	0.0496 (9)	
H8A	0.5599	1.1201	0.7425	0.074*	
H8B	0.6865	1.1277	0.5874	0.074*	
H8C	0.7678	1.0363	0.7222	0.074*	
Cl1	0.37658 (13)	0.35645 (9)	0.88661 (9)	0.0501 (2)	
Cl2	0.07883 (12)	0.68599 (9)	0.70197 (9)	0.0431 (2)	
N1	0.5878 (3)	0.6794 (3)	0.6377 (2)	0.0322 (6)	
N2	0.8705 (4)	0.8072 (3)	0.3791 (3)	0.0438 (7)	
N3	0.4131 (3)	0.8493 (3)	0.8015 (2)	0.0285 (5)	
N4	0.1750 (4)	0.6466 (3)	1.0283 (3)	0.0460 (7)	
H4A	0.0645	0.5900	1.0682	0.055*	
H4B	0.2545	0.6102	1.0879	0.055*	

### Atomic displacement parameters (Å^2^)

**Table:**

	*U*^11^	*U*^22^	*U*^33^	*U*^12^	*U*^13^	*U*^23^
Zn1	0.0306 (2)	0.0258 (2)	0.0363 (2)	−0.00363 (14)	−0.00618 (14)	−0.01066 (15)
C1	0.0433 (19)	0.0373 (19)	0.054 (2)	−0.0035 (15)	−0.0081 (16)	−0.0252 (16)
C2	0.047 (2)	0.057 (2)	0.047 (2)	0.0034 (17)	−0.0056 (16)	−0.0320 (18)
C3	0.0337 (17)	0.0355 (18)	0.0349 (16)	−0.0028 (13)	−0.0075 (13)	−0.0118 (14)
C4	0.0207 (14)	0.0295 (16)	0.0344 (16)	0.0014 (12)	−0.0101 (12)	−0.0130 (13)
C5	0.0256 (15)	0.0276 (16)	0.0315 (15)	0.0008 (12)	−0.0089 (12)	−0.0096 (13)
C6	0.0387 (17)	0.0318 (17)	0.0327 (16)	−0.0005 (13)	−0.0035 (13)	−0.0158 (13)
C7	0.0397 (19)	0.044 (2)	0.0408 (18)	−0.0042 (15)	0.0049 (14)	−0.0212 (16)
C8	0.049 (2)	0.046 (2)	0.055 (2)	−0.0185 (17)	0.0033 (17)	−0.0267 (17)
Cl1	0.0558 (6)	0.0267 (4)	0.0641 (6)	0.0026 (4)	−0.0167 (4)	−0.0108 (4)
Cl2	0.0419 (5)	0.0377 (5)	0.0505 (5)	−0.0055 (3)	−0.0203 (4)	−0.0077 (4)
N1	0.0312 (13)	0.0299 (14)	0.0373 (13)	−0.0033 (11)	−0.0055 (11)	−0.0150 (11)
N2	0.0402 (16)	0.0513 (18)	0.0404 (15)	−0.0058 (13)	−0.0001 (12)	−0.0225 (14)
N3	0.0296 (13)	0.0262 (13)	0.0313 (13)	0.0009 (10)	−0.0064 (11)	−0.0128 (11)
N4	0.0535 (18)	0.0400 (17)	0.0384 (15)	−0.0155 (14)	0.0007 (13)	−0.0111 (13)

### Geometric parameters (Å, °)

**Table:**

Zn1—N4	2.117 (3)	C5—N3	1.266 (3)
Zn1—N3	2.122 (3)	C5—C8	1.485 (4)
Zn1—N1	2.245 (2)	C6—N3	1.467 (3)
Zn1—Cl2	2.2807 (14)	C6—C7	1.510 (4)
Zn1—Cl1	2.2862 (16)	C6—H6A	0.9700
C1—N1	1.329 (4)	C6—H6B	0.9700
C1—C2	1.387 (4)	C7—N4	1.468 (4)
C1—H1	0.9300	C7—H7A	0.9700
C2—N2	1.320 (4)	C7—H7B	0.9700
C2—H2	0.9300	C8—H8A	0.9600
C3—N2	1.337 (4)	C8—H8B	0.9600
C3—C4	1.381 (4)	C8—H8C	0.9600
C3—H3	0.9300	N4—H4A	0.9000
C4—N1	1.335 (3)	N4—H4B	0.9000
C4—C5	1.505 (4)		
			
N4—Zn1—N3	77.98 (9)	N3—C6—H6B	110.0
N4—Zn1—N1	145.43 (10)	C7—C6—H6B	110.0
N3—Zn1—N1	73.32 (8)	H6A—C6—H6B	108.4
N4—Zn1—Cl2	104.77 (9)	N4—C7—C6	109.6 (2)
N3—Zn1—Cl2	104.26 (7)	N4—C7—H7A	109.8
N1—Zn1—Cl2	100.68 (8)	C6—C7—H7A	109.8
N4—Zn1—Cl1	100.56 (8)	N4—C7—H7B	109.8
N3—Zn1—Cl1	146.85 (7)	C6—C7—H7B	109.8
N1—Zn1—Cl1	93.29 (7)	H7A—C7—H7B	108.2
Cl2—Zn1—Cl1	108.03 (3)	C5—C8—H8A	109.5
N1—C1—C2	120.9 (3)	C5—C8—H8B	109.5
N1—C1—H1	119.5	H8A—C8—H8B	109.5
C2—C1—H1	119.5	C5—C8—H8C	109.5
N2—C2—C1	122.8 (3)	H8A—C8—H8C	109.5
N2—C2—H2	118.6	H8B—C8—H8C	109.5
C1—C2—H2	118.6	C1—N1—C4	117.2 (2)
N2—C3—C4	122.5 (3)	C1—N1—Zn1	128.1 (2)
N2—C3—H3	118.7	C4—N1—Zn1	113.90 (17)
C4—C3—H3	118.7	C2—N2—C3	115.7 (3)
N1—C4—C3	120.8 (2)	C5—N3—C6	122.9 (2)
N1—C4—C5	115.2 (2)	C5—N3—Zn1	121.79 (19)
C3—C4—C5	124.0 (3)	C6—N3—Zn1	114.89 (17)
N3—C5—C8	125.8 (3)	C7—N4—Zn1	106.93 (18)
N3—C5—C4	114.5 (2)	C7—N4—H4A	110.3
C8—C5—C4	119.6 (2)	Zn1—N4—H4A	110.3
N3—C6—C7	108.4 (2)	C7—N4—H4B	110.3
N3—C6—H6A	110.0	Zn1—N4—H4B	110.3
C7—C6—H6A	110.0	H4A—N4—H4B	108.6
			
N1—C1—C2—N2	0.9 (5)	C1—C2—N2—C3	1.6 (5)
N2—C3—C4—N1	−1.1 (4)	C4—C3—N2—C2	−1.5 (4)
N2—C3—C4—C5	179.4 (3)	C8—C5—N3—C6	−1.6 (5)
N1—C4—C5—N3	−11.6 (4)	C4—C5—N3—C6	176.5 (2)
C3—C4—C5—N3	167.8 (3)	C8—C5—N3—Zn1	−173.7 (2)
N1—C4—C5—C8	166.6 (3)	C4—C5—N3—Zn1	4.4 (3)
C3—C4—C5—C8	−13.9 (4)	C7—C6—N3—C5	173.4 (3)
N3—C6—C7—N4	43.0 (3)	C7—C6—N3—Zn1	−13.9 (3)
C2—C1—N1—C4	−3.5 (5)	N4—Zn1—N3—C5	162.1 (2)
C2—C1—N1—Zn1	165.3 (2)	N1—Zn1—N3—C5	1.6 (2)
C3—C4—N1—C1	3.6 (4)	Cl2—Zn1—N3—C5	−95.5 (2)
C5—C4—N1—C1	−176.9 (3)	Cl1—Zn1—N3—C5	71.1 (3)
C3—C4—N1—Zn1	−166.8 (2)	N4—Zn1—N3—C6	−10.6 (2)
C5—C4—N1—Zn1	12.7 (3)	N1—Zn1—N3—C6	−171.1 (2)
N4—Zn1—N1—C1	147.8 (3)	Cl2—Zn1—N3—C6	91.80 (19)
N3—Zn1—N1—C1	−177.1 (3)	Cl1—Zn1—N3—C6	−101.6 (2)
Cl2—Zn1—N1—C1	−75.2 (3)	C6—C7—N4—Zn1	−51.8 (3)
Cl1—Zn1—N1—C1	33.8 (3)	N3—Zn1—N4—C7	33.3 (2)
N4—Zn1—N1—C4	−43.1 (3)	N1—Zn1—N4—C7	67.6 (3)
N3—Zn1—N1—C4	−8.02 (18)	Cl2—Zn1—N4—C7	−68.5 (2)
Cl2—Zn1—N1—C4	93.82 (19)	Cl1—Zn1—N4—C7	179.54 (19)
Cl1—Zn1—N1—C4	−157.14 (19)		

### Hydrogen-bond geometry (Å, °)

**Table:**

*D*—H···*A*	*D*—H	H···*A*	*D*···*A*	*D*—H···*A*
N4—H4B···Cl1^i^	0.90	2.77	3.531 (4)	143.
N4—H4A···Cl2^ii^	0.90	2.81	3.534 (3)	139.
C2—H2···Cl2^iii^	0.93	2.81	3.672 (4)	155.
C3—H3···Cl2^iv^	0.93	2.80	3.704 (4)	164.
C6—H6B···Cl1^i^	0.97	2.84	3.550 (4)	131.

Symmetry codes: (i) −*x*+1, −*y*+1, −*z*+2; (ii) −*x*, −*y*+1, −*z*+2; (iii) −*x*+1, −*y*+1, −*z*+1; (iv) −*x*+1, −*y*+2, −*z*+1.
